# The Case of John Jolly

**Published:** 1884-01-20

**Authors:** A. W. Reese

**Affiliations:** Warrensburg, Missouri


### THE CASE OF JOHN JOLLY.

BY A. W. REESE, M. D., OF MISSOURI.

I wish to report, for the readers of the SOUTHERN MEDICAL RECORD, a remarkable surgical case that occurred, under my notice, during the late civil war.

In August, 1864, that part of the U. S. Army commanded by Major Gen. Wm. T. Sherman occupied the trenches in front of Atlanta.

The writer was, at that time, connected with the medical staff of that army, being Surgeon of the 31st Mo. Infantry, commanded by ex-Gov. Thos. C Fletcher.

Without entering into the details of that memorable siege, I cannot refrain from presenting one of its thrilling incidents, which includes the above-mentioned case.

It will be remembered that, after the sanguinary engagements of the 20th of July on Peachtree Creek, and the 22d and 28th in front of Atlanta, the military operations of the Federal Army were confined to the monotonous duties of a siege, relieved by an occasional "lively time" on the skirmish line, fragmentary cavalry raids, or an "artillery duel" between the opposing forces.

A part of this time the author was Brigade Surgeon of the 3d Brigade, 1st Division, 15th Army Corps (John A. Logan's), and, while serving in that officail capacity, the case spoken of came under his notice.

Orders were received, one evening, about sun-down, for the 3d Brigade to move to the front, for the purpose of advancing our skirmish line.

It was a perilous duty, for we were well aware of the stubborn resistance to be met. We had seen, on many hotly contested fields, the metal of the Confederate veterans commanded by that able General, Jos. E. Johnston.

We moved out, silently and cautiously, to the front—all the officers, field and staff, being dismounted.

Reaching the summit of a small woodland crest, the 3d Brigade formed in line of battle, and the bugles immediately sounded the charge. Simultaneously with the advance, one of our 30 ℔ Parrot batteries opened its thunders on the enemy's works, to cover and protect the movement of the troops.

Before the fierce, determined impetuosity of this charge, the Confederate skirmish line was driven back, and the Brigade took possession of the abandoned pits.

But the advantage gained proved but a temporary success. In a moment the batteries of the enemy opened on the Brigade, and, amid a perfect tempest of shot and shell, our forces were compelled to retreat.

The medical officers of the Brigade had but imperfect shelter from this rough storm of shell, cannister and grape, that literally rained down upon us.

We sought refuge in some abandoned skirmish pits, and dressed the wounds of the men brought in, on our knees. At length, under "the leaden rain and iron hail" from the enemy's guns, we were compelled to lie down flat on the bottom of the trenches and temporarily suspend our surgical procedures for the relief of the wounded men.

We were soon covered with leaves, twigs and boughs of trees, cut to shreds by the flying missiles overhead.

To add still further to the personal discomfort of the medical staff, heavy masses of dark clouds, which had begun to loom up on the horizon's bar, now drifted rapidly to that portion of the sky immediately above us, and, while the thunders above added their sublime voice to the terrific roar of the enemy's cannon, the *rain* began to pour down, as it only *can* pour down in a rain-storm of Northern Georgia.

We were very soon drenched to the skin. But presently came the order to retreat. This was done in some confusion, and under fire. A major of infantry, immediately in front of the writer, was struck on the head by a flying fragment of shell, and instantly killed.

The explosion of a second shell, in our ranks, killed six men and wounded a number of others—among these latter, John Jolly, private of Co. C. of my own regiment, whose case forms the principal subject of this paper.

He was picked up by two comrades, and borne along in the retreat.

When the command had reached the cover of a small hill, and a halt made, this wounded soldier was brought to me for aid. He was a heavy-set, dark-haired, olive-complexioned man, with a decided *French* aspect; about thirty years old.

His right arm was frightfully mangled at the elbow joint; the brachial artery was completely severed; the hemorrhage had been profuse. He was in a state bordering on collapse. The skin was cold, and covered with a clammy sweat. The pulse had vanished from the right wrist, and was small, intermittent and thready in the left. The respiration was bad, and the face cadaverous, ghastly and white. In short, the symptoms were those of impending dissolution.

A glass of whisky was immediately administered; I watched him narrowly, and as soon as the pulse in the left wrist and a slight flush on the countenance indicated some response to the stimulant, secured the brachial artery and dressed the shattered elbow. As it was now quite dark, and further surgical interference under the surrounding circumstances out of the question, he was put into an ambulance and sent back to the division field hospital, one mile to the rear.

I might here state that I observed, during the primary examination of this man, what seemed to be a slight flesh wound on the right side of the chest, between the 6th and 7th ribs. In the meantime the brigade had formed in line and marched back to its intrenchments in the reserve rifle-pits.

The morning succeeding the events just narrated, when freed from official duty at camp, I mounted my horse and rode out to the division field hospital, which was under the care of Surgeon B. N. Bond, of the 29th Missouri Infantry.

I spent a half hour at Surgeon Bond's tent. In the course of the interview he inquired about the engagement of the previous evening, presuming that my duties would call me to the field.

After a brief statement of the affair, on my part, he remarked:

"A very singular case was brought in from that fight last night; a man with his right arm shattered at the elbow and a wound in the side. He belongs to your regiment, I think!"

"I remember the man," said I, "for he was brought to me on the field, but I failed to see anything of special *professional* interest in the case."

Bond arose, went to a small valise, opened it, took something out, and turning to me said:

"What do you call that?"

It was a piece of iron about the size of the palm of a man's hand; an irregular circle in shape, convex on one side, and concave on the other, and about one inch thick. Its edges were serrated and rough, and its weight, at a moderate estimate, two pounds.

"I call that," said I, "a pretty liberal fragment of a shrapnell shell! What about it?"

"What would you say," remarked Bond. "if I were to tell you that I removed that piece of pot-metal last night, from the chest of the man in question, where it had been driven with great violence and deeply imbedded in the lung?"

"It would be bad for the man," said I.

"Well, that is what I did last night, after amputation of the arm above the elbow joint."

"Has the man been buried yet?"

Surgeon Bond gave me a quick, searching look, and answered dryly:

"Well, no. We don't usually *bury* men, even down here, *till they're dead!*"

"He is then, *still alive?*"

"He *is*—or was, at least, about an hour ago! Would you like to see him?"

"By all means in the world!"

As we walked across the hospital grounds, toward the surgical ward, Bond gave me the details of the case.

After amputating the arm, and while the patient was yet profoundly under the influence of chloroform, Dr. Bond discovered the small wound on the right side of the chest.

He, at first, like myself, supposed it to be merely a slight flesh wound, but on further examination found it to communicate with the cavity of the chest. The probe also revealed the presence of a *metallic* substance embedded in the lung.

A consultation, comprising the entire medical staff of the field hospital, was at once held. The opinions were varied and somewhat conflicting, but it was finally agreed upon to attempt the removal of the foreign body from the chest.

In effecting this the orifice of entrance had to be enlarged three inches, and it required considerable force to dislodge the missile from its bed. There was but little hemorrhage during the operation, the pulse remained good, and on his emergence from the influence of the chloroform, the patient was given one grain of the sulphate morphia and put to bed.

We passed into the long, white row of hospital tents filled with the wounded men. We passed down the "long drawn aisle" bordered with pale and ghastly faces, till we came to the couch where the wounded man, Jolly, lay. He was still asleep. His respiration was regular. soft and noiseless. He was very pale. I put my fingers on the remaining wrist, and took out my watch. While I was thus engaged he suddenly awoke. There was the light of intelligence in his eyes.

"Well, my man," said Bond, "how do you feel, to-day?"

"Pretty well, Doctor; I don't *feel any pain*, but I'm *very weak!*"

"Who is that?" asked Bond, pointing to me.

"Why," said Jolly, seemingly surprised at the question, "that's *our Surgeon*, Doctor Reese!"

After some further conversation with Jolly, and looking through the wards with Surgeon Bond, I remounted my steed and returned to our encampment at the front.

This case was so remarkable, and so full of professional interest, that I went out every day to the division hospital to note its progress and final result.

He remained in the first division field hospital, in front of Atlanta, about two weeks, and steadily improved during the whole of that time.

When I saw him last his condition was good, and the prospects of his ultimate recovery quite fair. His pulse was 80, full, round and soft; his skin was moist and pleasant to the touch; the bowels regular, tongue clean, appetite good, and he slept well at night, without an opiate.

There had been no secondary hemorrhage; there was but little cough, and the wound in the chest was discharging moderately a healthy pus.

On the night succeeding my last visit to Jolly, Gen. Sherman instituted his celebrated "flank movement" on the railway connections Southwest of Atlanta. Orders were issued to all the medical officers of his army to send the sick and wounded to the rear.

In accordance with these orders Jolly was put into an ambulance, jolted over a miserable road, cut into ruts and "chuck holes" by supply, ordinance and artillery trains, a terrible journey for such a man—back to Marietta, a distance of 18 miles, and assigned to the corps hospital at that place. I regret to add that he perished from secondary hemorrhage, shortly after his arrival at the hospital in Marietta.

I am fully convinced that if the premature removal of this case could have been avoided, if he could have been kept in the division hospital near Atlanta a few weeks longer, this soldier, terrible as his injuries were, would have ultimately recovered. As it is, however, the case is most instructive, and one of the most remarkable in the annals of military surgery.

*Warrensburg, Missouri.*

---

**Ether in Typhoid Fever.**—A French physician considers hypodermic injections of ether very valuable in the adynamic forms of the disease. He reports five cases so treated. Two injections of twenty drops each time, were made daily, and under its influence the patient was aroused and delirium ceased. In pneumonia these injections are of the greatest utility, as they are in every malady assuming a typhoid form.—*Louisville Med. News.*

